# Prophylactic vs preemptive strategy for the prevention of CMV disease in solid organ transplant recipients: systematic review and meta-analysis of randomized controlled trials

**DOI:** 10.1007/s15010-024-02441-4

**Published:** 2024-11-22

**Authors:** Niv Reiss-Gindi, Tomer Hoffman, Tanya Ruderman, Alaa Atamna, Ili Margalit, Dafna Yahav

**Affiliations:** 1 Medicine D, Sheba Medical Center, Ramat-Gan, Israel; 2 Infectious Diseases Unit, Sheba Medical Center, 2 Sheba Road, 52621 Ramat-Gan, Israel; 3 Medicine A, Sheba Medical Center, Ramat-Gan, Israel; 4 Infectious Diseases Unit, Rabin Medical Center, Beilinson Hospital, Petah-Tikva, Israel; 5 Faculty of Medicine, Tel-Aviv University, Ramat-Aviv, Tel-Aviv, Israel

**Keywords:** Solid organ transplant, CMV, Prophylaxis, Preemptive, Mortality

## Abstract

**Purpose:**

Cytomegalovirus (CMV) is associated with significant morbidity and mortality among solid organ transplant (SOT) recipients. Strategies for CMV prevention include universal prophylaxis or preemptive approach. We aimed to evaluate the optimal approach.

**Methods:**

We performed a systematic review and meta-analysis of randomized controlled trials comparing prophylaxis versus preemptive therapy for CMV in SOT. The primary outcome was CMV disease. Subgroup analysis of outcomes in D+ R− patients was performed.

**Results:**

Nine trials have met inclusion criteria, five of them included kidney transplant recipients, all compared val/ganciclovir universal prophylaxis versus preemptive approach. Universal prophylaxis resulted in lower probability of CMV infection (relative risk [RR] 0.44, 95% confidence interval [CI] 0.33–0.58), yet the impact on CMV disease was insignificant (RR 0.54, 95% CI 0.24–1.23), in neither SOT recipients in general nor among D+R− subgroup (RR 0.93, 95% CI 0.37–2.32). Late-onset CMV disease rates were lower with preemptive approach. Sensitivity analysis according to allocation concealment and blinding showed similar results for CMV disease. No significant differences were demonstrated for the outcomes of mortality, bacterial or fungal infection or graft related outcomes. Acute kidney injury was significantly more common with prophylaxis (RR 1.79, 95% CI 1.12–2.89).

**Conclusion:**

Preemptive approach is a reasonable approach for CMV prevention in SOT recipients, if feasible. Strategies for combining the preemptive with prophylaxis strategies, as well as immune monitoring, should be investigated.

**Supplementary Information:**

The online version contains supplementary material available at 10.1007/s15010-024-02441-4.

## Introduction

Cytomegalovirus (CMV) is a significant cause of morbidity and mortality among solid organ transplant (SOT) recipients. Seronegative recipients of seropositive donors (D+/R−) are considered at highest risk for CMV disease. CMV prevention in transplant recipients is achieved through either universal prophylaxis or preemptive therapy. Universal prophylaxis is implemented through administration of antiviral therapy for a predefined period to all SOT recipients. The preemptive strategy is carried out by periodic CMV viremia monitoring using serial PCR-based assays to detect viral replication and provide early treatment to prevent CMV disease [1, 2].

Two meta-analyses of randomized controlled trials (RCTs) conducted over a decade ago demonstrated benefits of prophylaxis over preemptive approach, with successful CMV disease prevention, as well as better prevention of other herpes viruses, bacterial and fungal infections. Yet, both meta-analyses reached contradictory results regarding the impact of CMV prophylaxis on overall mortality [1, 2]. A more recent meta-analysis of studies of any design focused on D+R− kidney transplant recipients, and demonstrated no mortality benefit [3]. The major disadvantages of universal prophylaxis are drug toxicity, including leukopenia and neutropenia, and late-onset CMV disease. By contrast, with the preemptive approach, antivirals are less often prescribed and delayed-onset CMV disease has been reported to be less common [4, 5].

We performed an updated systematic review and meta-analysis of randomized controlled trials (RCTs) assessing the efficacy of universal prophylaxis *vs* preemptive approach for CMV prevention among SOT recipients.

## Materials and methods

We conducted a systematic review in alignment with the PRISMA guidelines [6]. The study protocol was registered in PROSPERO (CRD42023398555).

### Types of studies, participants, interventions and comparisons

We included RCTs evaluating preemptive approach compared with universal antiviral prophylaxis in SOT recipients. Search was conducted until 28 Jan 2024, and was not limited by year of publication, language, or publication status (published articles, conference proceedings or unpublished).

Patients included are adult SOT recipients, immediately following transplantation of any solid organ (single or multiple), with any CMV serostatus of both recipient and donor. We compared preemptive approach, regardless of CMV PCR monitoring schedule, *vs* antiviral prophylaxis (see definitions below) with an anti-CMV agent, including ganciclovir, valganciclovir, foscarnet, cidofovir, letermovir or maribavir. Trials evaluating prophylaxis with acyclovir or valacyclovir were excluded, as these drugs are used for the prevention of other herpesviruses while practicing the preemptive approach [7].

### Type of outcome measures

The primary outcome was CMV disease as defined below, during the 12 months post-transplant.

Secondary outcomes, as defined below, at 3, 6, and 12 months, included (if available): CMV disease at 3 and 6 months, late-onset CMV disease, as defined in individual studies, CMV infection, as defined below, all-cause mortality, graft loss and acute rejection, as defined in individual studies, bacterial or fungal infections, time to development of CMV infection or disease, adverse events (neutropenia [defined as absolute neutrophil count below 500 cells/mm [3]], thrombocytopenia, acute kidney injury as defined in individual studies, any serious adverse events), malignancy, quality of life, and Human herpes virus 6 (HHV-6) reactivation.

### Definitions

Preemptive approach was defined as routine PCR testing for CMV viremia and initiating antiviral treatment if viremia is present, with frequency of testing, cut-off for treatment, and therapeutic regimens as defined in individual studies.

Universal prophylaxis was defined as routine administration of an anti-CMV agent for a pre-specified period early post transplantation.

CMV Infection and CMV disease, including specific patterns, were defined as accepted in international guidelines for CMV in SOT recipients [7–9].

### Search

We searched the following electronic databases since conceived until 28 Jan 2024: PubMed, The Cochrane Central Register of Controlled Trials (CENTRAL, the Cochrane Library), Web Of Science, as well as the European Congress of Clinical Microbiology and Infectious Diseases (ECCMID) (2012–2023), and ID week 2018 to 2023. Reference lists of all included studies were scanned for additional relevant trials. Search terms for each database are detailed in the Supplemental appendix.

### Study selection

One reviewer (DY) performed the search and inspected the title and if needed, the abstract of each reference identified. Where relevant articles were identified, the full article was obtained and inspected independently by two reviewers (DY, NRG, TH, AA, IM) who applied inclusion and exclusion criteria.

### Data collection

Two reviewers (DY, NRG, TR, TH( independently extracted data from included studies into a data extraction sheet. Differences in the data extracted were discussed and if still agreement was not achieved, resolved by discussion with a third reviewer. Justification for excluding studies from the review were documented. The first or corresponding author of each included study was contacted for clarifications and further information, if needed. Outcomes were extracted, preferably by intention-to-treat, and if not available, by per protocol population.

### Quality assessment of studies

Two reviewers independently assessed the risk of bias in included trials according to the domain-based evaluation recommended by the Cochrane handbook [10]. Each domain was assigned low, high, or unclear risk of bias, using the definitions provided in the Cochrane handbook. The domains evaluated included allocation generation, allocation concealment, blinding, incomplete outcome data, and selective outcome reporting.

### Statistical analysis

The meta-analysis was conducted using the Cochrane Review Manager (RevMan) Web. We compiled relative risks (RRs) with 95% confidence intervals (CIs) of individual trials using random-effects model. Heterogeneity was assessed using a Chi-square test of heterogeneity and the I^2^ measure of inconsistency [11]. Substantial heterogeneity was considered as I^2^ > 50%. Assessment of reporting bias was planned to be conducted via funnel plot in order to assess small study effects, though eventually less than 10 trials were included per analysis and funnel plots were not created. Forest plots were primarily sub-grouped by type of organ transplanted. Sensitivity analyses were planned according to allocation concealment risk of bias. Subgroups analyses were pre-planned in accordance to D+R− status, thoracic organ transplant (lung, heart, or combined), and long-term prophylaxis (> 6 months). The two latter subgroups were eventually unavailable for this meta-analysis.

## Results

**Fig. 1:**
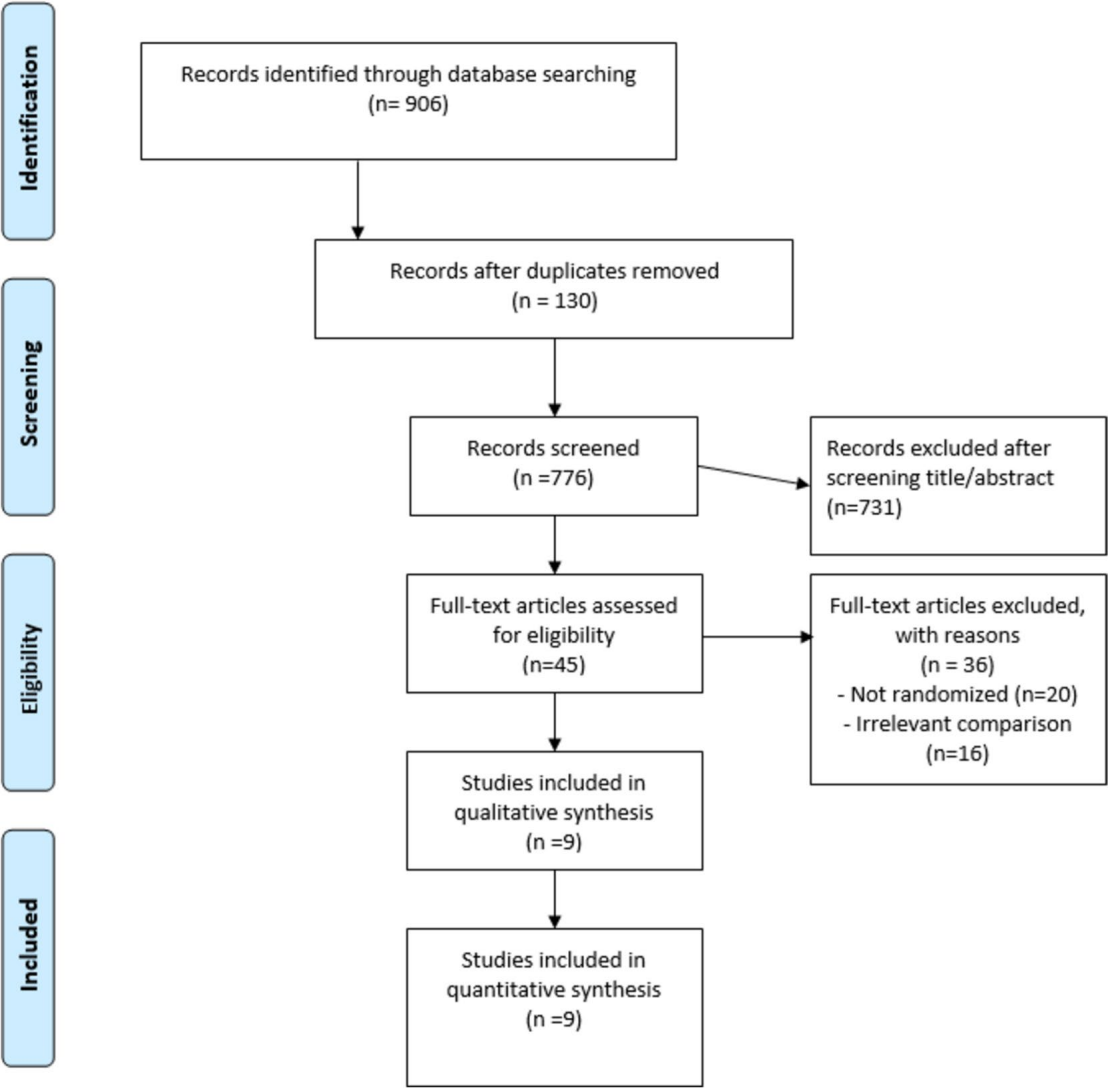
Study flowchart

The study flow chart is presented in Fig. 1 (according to PRISMA flow diagram). 45 publications were retrieved for full-text assessment, nine of them (comprising 1439 SOT recipients) met inclusion criteria and were included in the quantitative meta-analysis. One publication reported post hoc long-term results of the trial by Singh et al. and was not included in the analysis [12].

Six trials used prophylaxis with valganciclovir and three with oral ganciclovir [13–15]. All but 2 trials [16, 17] included D+R− population, and one trial [18] focused on D+R- patients only. Table 1 reports the characteristics of trials included in the meta-analysis. Table 2 details prophylaxis and preemptive regimens and timeframes. All included trials prescribed prophylaxis for ~ 3 months. Seven out of nine trials implemented preemptive approach for 12 months, while two of the trials practiced the approach for either 9 months (following an initial 3 months period of prophylaxis) [19] or 18 months. [17]

**Table 1:** Characteristics of included trials

Trial ID	Type of organ	Type of patients included	No randomized	Antiviral prophylaxis	Antiviral preemptive
Reischig 2023 [27]	Kidney	**D+R−,** D+R+, D−R+	140	70	70
Singh 2020 [18]	Liver	**D+R−**	205	105	100
Witzke 2012 [16]	Kidney	D+R+, D−R+	297^a^	146	150
Scott 2011 [17]	Liver	D+R+, D-R+, D−R−	49	24	25
Palmer 2010 [19]	Lung	**D+R−**, D+R+, D−R+	136	70	66
Kliem 2008 [15]	Kidney	**D+R−**, D+R+, D−R+	148	74	74
Khoury 2006 [4]	Kidney	**D+R−**, D+R+, D-R+	98	50^b^	49
Jung 2001 [14]	Kidney	**D+R−**, D+R+, D−R+	70	34	36
Gane 1997 [13]	Liver	**D+R−**, D+R+, D−R+	304	150	154

^a^ One patient did not receive study medication following randomization and was excluded from the analysis

^b^ One patient in the prophylactic group had primary nonfunction within the first week and was excluded from the analysis

**Table 2:** Prophylaxis and preemptive management in included trials

Trial ID	Duration of prophylaxis	Monitoring in prophylaxis arm	Cutoff for preemptive therapy	Duration of preemptive	Frequency of monitoring	Timing of CMV disease follow up	Late-onset definition
Reischig 2023	3 m (6 m D+R−)	Y	≥ 1000 IU/mL	12 m	Weekly for 4 M, monthly 4–12 M	12 m	> 3 m
Singh 2020	100d	N	Any level	12 m	Weekly	12 m	3–12 m
Witzke 2012	100d	UN	≥ 400 copies/mL	12 m	1–4 W—weekly, 5–28 W—Every 3W, 29–52 W—Every 3M, then Every 6 M	12 m	> 3 m
Scott 2011	100d	Y	NS	18 m	1–12 W—weekly, then Monthly	18 m	None
Palmer 2010	12m*	Y	NS	9 m (after 3 m prophylaxis)	0–6 M—Every 2 weeks, then Monthly	13 m	> 12 m
Kliem 2008	3m	Y	≥ 400 copies/mL	12 m	W1–4—Weekly, W5–12—every 2 W, W13–24—monthly, W25–52—Every 3 M	12 m	None
Khoury 2006	100d	Y	≥ 2000 copies/mL	12 m	1–16 W—Weekly, 5, 6, 9 and 12 months	12 m	3–12 m
Jung 2001	3m	Y	≥ 400 copies/mL	12 m	0–1 M—twice weekly, 2–3 M—weekly, 4–5 M—twice monthly, 6–12 M—monthly	12 m	NS
Gane 1997	97d	Y	NS	12 m	Days 10, 14, 21, 28, 42, 56, 70, 98, M4, M5, M6, M12	6 m	3–6 m

^a^ Both groups received prophylaxis for 3 months and then randomized for 9 more months for extended prophylaxis vs preemptive

*UN* unknown

**Fig. 2:**
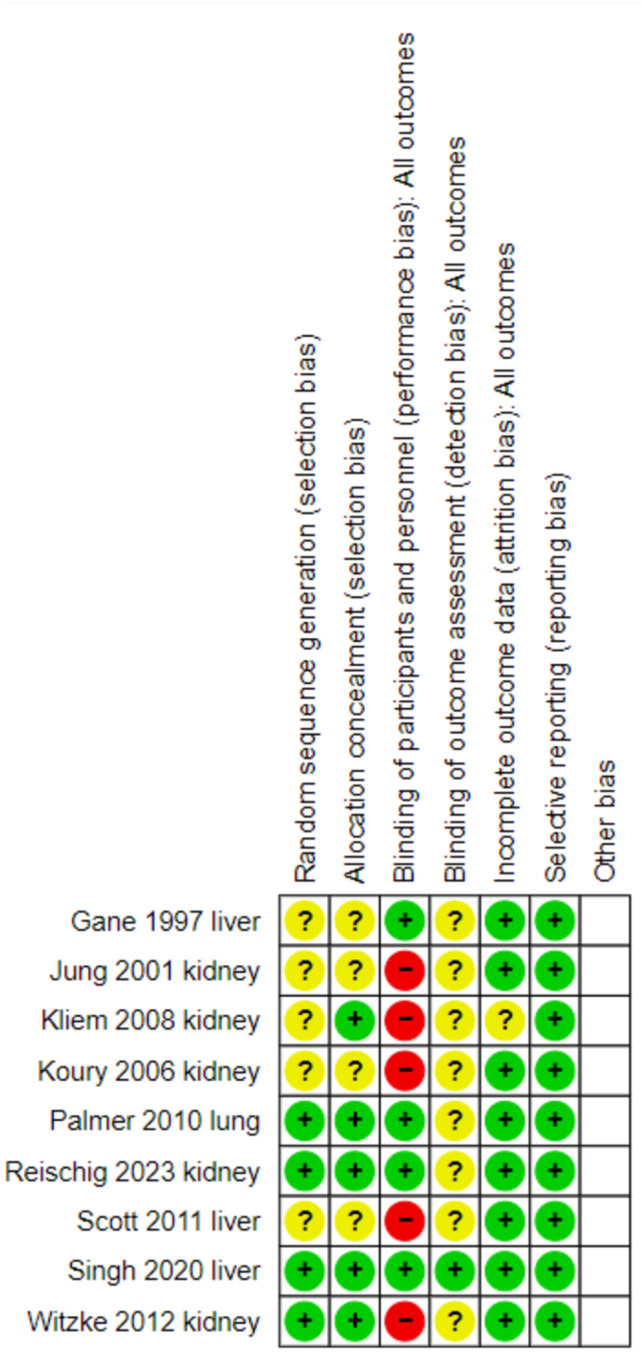
Risk of bias assessment

Of the nine included publications, four were considered as having low risk of bias for allocation generation, five for allocation concealment, and four were double blinded. (Fig. 2).

### ***CMV disease (***Fig. 3***)***

**Fig. 3:**
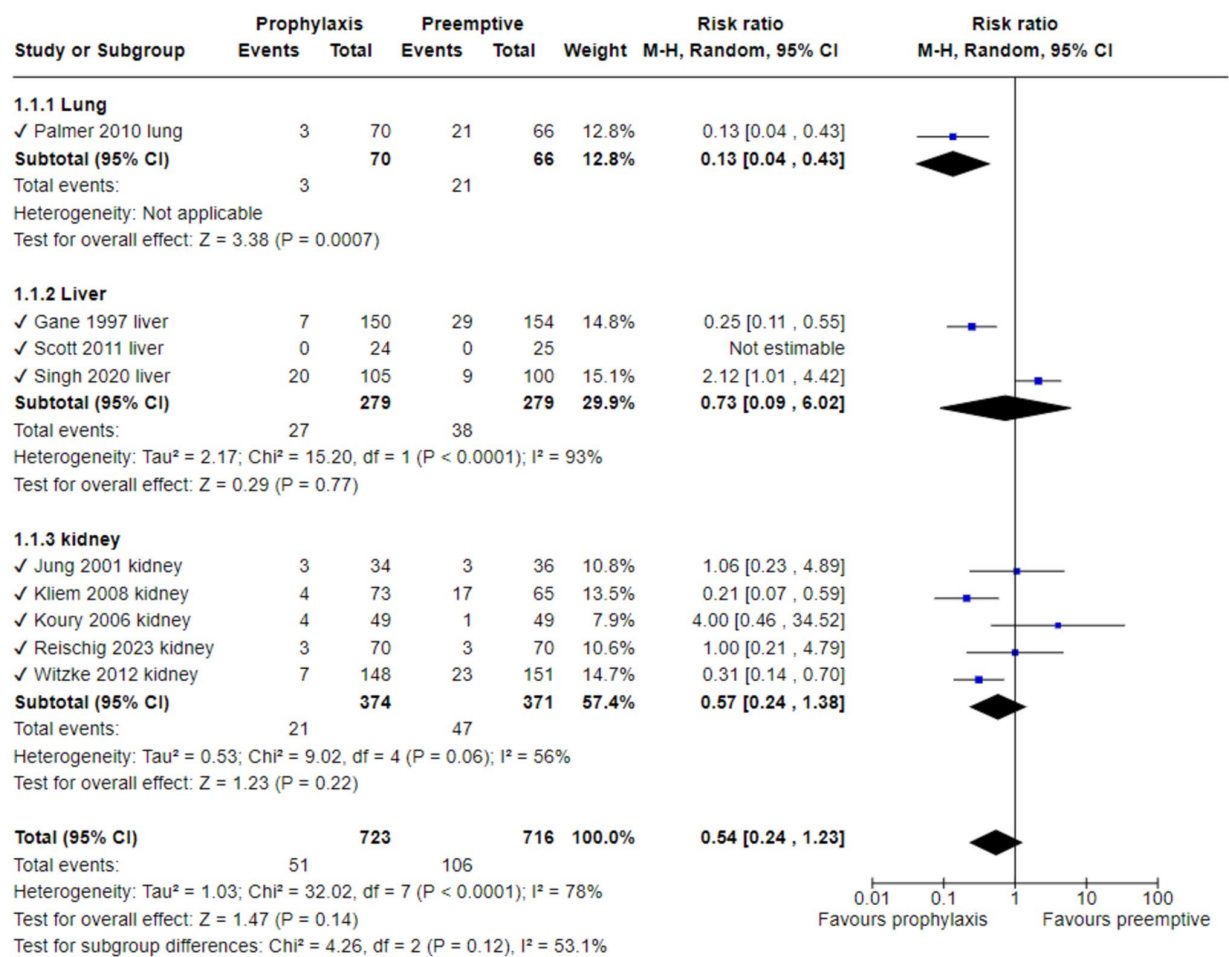
CMV disease

Nine studies reported the primary outcome, CMV disease at 12 months: one reported lung transplant recipients (LuTR), five kidney transplant recipients (KTR), and 3 liver transplant recipients (LTR). Overall, no significant difference in CMV disease rates were demonstrated between groups (RR 0.54, 95% CI 0.24–1.23, with substantial heterogeneity, I^2^ = 78%). (Fig. 3) Excluding one trial [18] (Singh et al.) resulted in significant results favoring prophylaxis (RR 0.38, 95% CI 0.20–0.73), while reducing heterogeneity to I^2^ = 53%.

Sensitivity analyses were performed for allocation concealment and blinding. Among 5 trials with low risk of bias for allocation concealment and 4 double blind trials, no significant difference between study groups was demonstrated (RR 0.45, 95% CI 0.15–1.27, and RR 0.52, 95% CI 0.13–2.05, respectively). Additional sensitivity analysis excluding 3 trials using oral ganciclovir as prophylaxis also demonstrated no difference (RR 0.72, 95% CI 0.22–2.46).

CMV gastrointestinal disease was assessed in 5 trials (2 of them reporting no cases), demonstrating no significant difference (RR 0.36, 95% CI 0.08–1.57, I^2^ = 0); CMV pneumonia was assessed in four trials, while only one trial reported cases and showed no significant difference between arms (RR 0.11, 95% CI 0.01- 2.10). CMV hepatitis was assessed in six trials; only two reported cases, with no significant difference between arms and conflicting results in these two trials (RR 0.61, 95% CI 0.02–15.28). CMV syndrome was assessed in eight trials, with no significant difference between arms (RR 0.56, 95% CI 0.23–1.35, I^2^ = 56%). Excluding the trial by Singh et al. resulted in significantly lower rates of CMV syndrome with prophylaxis, with reduced heterogeneity (RR 0.39, 95% CI 0.18–0.83, I^2^ = 38%). (See Supplemental Fig. 1).

**Fig. 4:**
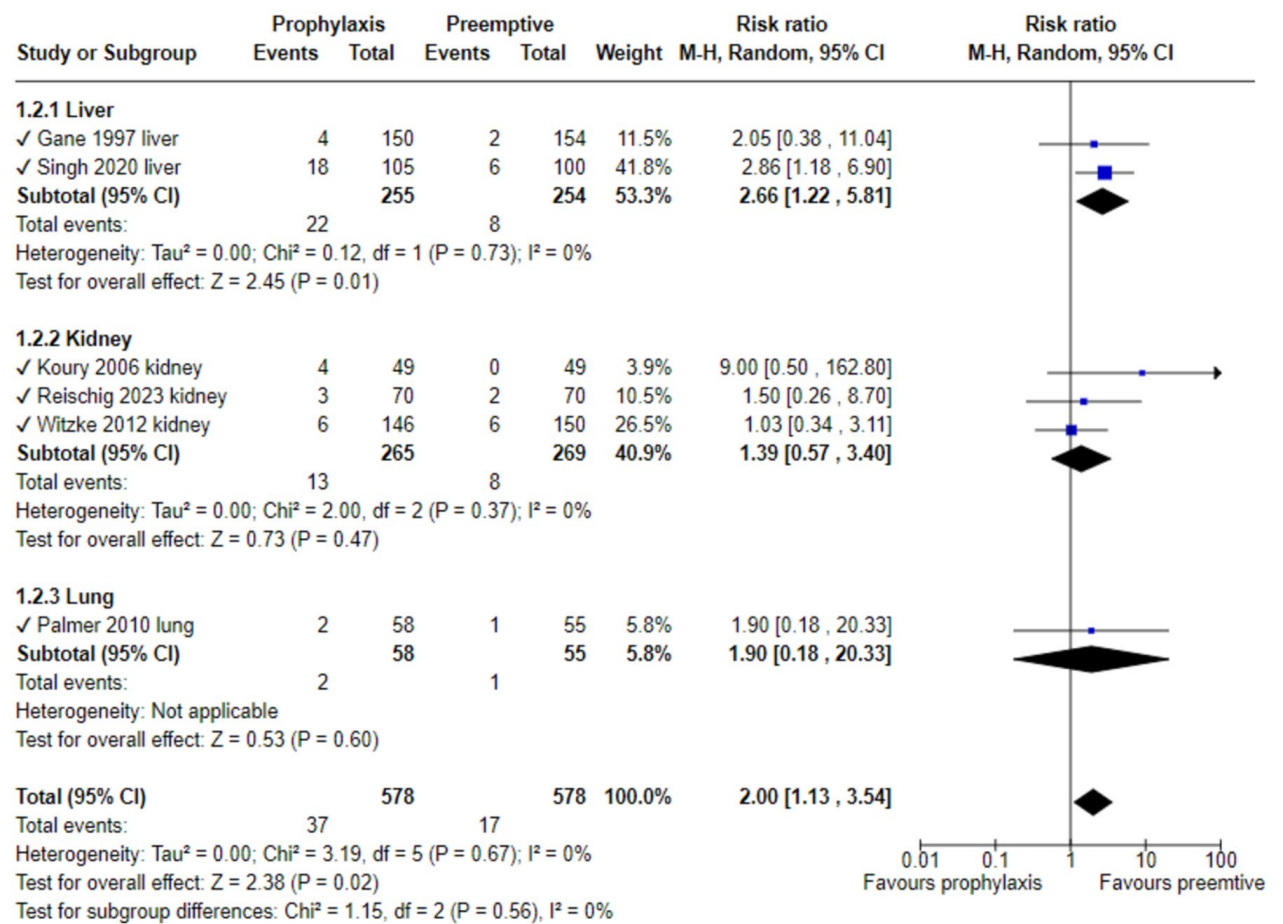
Late CMV disease

Late-onset CMV disease was reported from six trials (2 LTR, 3 KTR, 1 LuTR), showing significantly reduced CMV disease rates with preemptive therapy (RR 2.0, 95% CI 1.13–3.54, without heterogeneity, I^2^ = 0). (Fig. 4) In these trials prophylaxis was administered for 3 months in all, and late-onset disease was reported between 3 to 12 months, except Gane et al. [13] reporting between 3 months until 6 months, and Palmer et al. [19] for > 12 months.

### CMV infection

CMV infection at 12 month was reported from eight trials (4 KTR, 2 LTR, 1 LuTR). As expected by the intervention, infection rates were significantly lower in the prophylaxis arm, RR 0.44, 95% CI 0.33–0.58, I^2^ = 68%. (Fig. 4) Heterogeneity stemmed from differences in the size of the effect rather than the direction of the effect. Late-onset CMV infection was reported from 5 trials, without significant difference between arms (RR 2.77, 95% CI 0.91–8.45, I^2^ = 72%).

### Other outcomes

Overall mortality was reported in eight trials (4 KTR, 2 LTR, 1 LuTR), with no significant difference between preemptive approach and prophylaxis (RR 0.96, 95% CI 0.64–1.43, without heterogeneity). (See Supplemental Fig. 2).

Graft related outcomes were demonstrated without significant differences between arms (graft loss, RR 0.75, 95% CI 0.42–1.36, without heterogeneity, seven trials; graft rejection, RR 0.85, 95% CI 0.70–1.04, I^2^ = 18%). (See Supplemental Fig. 3 and 4, respectively).

Bacterial and fungal infection rates also did not differ between preemptive approach and prophylaxis (bacterial infections, RR 0.97, 95% CI 0.78–1.20, without heterogeneity, four trials; fungal infections, RR 1.28, 95% CI 0.55–3.00, three trials, I^2^ = 29%). Malignancy was reported in only two trials, showing no significant difference between arms (RR 0.89, 95% CI 0.50–1.58, without heterogeneity). Infection with HHV-6 and quality of life were not reported in any of the trials.

Time to CMV disease/infection was significantly longer in the prophylaxis arm (standard mean difference 9.94 months, 95% CI 5.82–14.06).

### Subgroup analysis

CMV disease among D+R− participants was reported in five trials, showing no significant difference between arms, RR 0.93, 95% CI 0.37–2.32, I^2^ = 54%. Only two trials reported CMV infection among D+R−, with no difference between prophylaxis and preemptive approach (RR 0.58, 95% CI 0.27–1.25).

### Adverse events

Adverse events requiring discontinuation of study drug were reported in four trials without significant difference (RR 1.34, 95% CI 0.70–2.54). Serious adverse events were also reported from 4 trials without difference (RR 0.98, 95% CI 0.83–1.16). Neutropenia (six trials) and thrombocytopenia (three trials) occurred without significant difference between study arms (RR 1.17, 95% CI 0.75–1.83 and RR 1.09, 95% CI 0.83–1.45, respectively). Acute kidney injury was significantly more common with prophylaxis (RR 1.79, 95% CI 1.12–2.89).

## Discussion

In this meta-analysis of nine randomized controlled trials (1439 patients), CMV disease after solid organ transplantation was not significantly different between prophylaxis (usually administered for ~ 3 months) or preemptive approach. Late-onset CMV disease was significantly lower in the preemptive arm.

The heterogeneity demonstrated in the results may be explained by the various organ transplant types included and the different CMV risk categories. Exclusion of the recent trial of D+R− liver transplant recipients by Singh et al. reduced heterogeneity and changed results to significantly favoring prophylaxis for preventing CMV disease. This trial used the lowest viral load cutoff for starting therapy (any level) and the strictest monitoring (weekly for 12 months) (See Table 2). No significant difference in overall mortality was demonstrated between groups. Assessing the subgroup of patients at highest risk for CMV (D+R− baseline serology), no significant differences between prophylaxis and preemptive were demonstrated for the outcomes of CMV disease and CMV infection.

No difference between arms was demonstrated for other types of infection, including bacterial and fungal infection, or for malignancy. Graft loss and graft rejection rates were also without significant difference. In terms of adverse events, no difference in either thrombocytopenia or neutropenia was observed, serious adverse events and those requiring discontinuation of study were also not significantly different. Nevertheless, acute kidney injury was significantly more common with prophylaxis.

Previous meta-analyses of RCTs addressing this question were published over a decade ago. Kalil et al. in 2005 found that universal prophylaxis reduced CMV disease among the D+R− group and also reduced bacterial and fungal infections, as well as mortality [1]. A later meta-analysis by Owers et al. found no significant difference in neither CMV disease rates between prophylaxis and preemptive approach, nor in mortality and other infections [2]. Kumar et al. preformed a recent meta-analysis including studies of any design, including non-comparative studies, that evaluated prophylaxis and/or preemptive therapy among D+R− kidney transplant recipients. In this meta-analysis, studies using preemptive therapy for low threshold CMV (~ 100 IU/ml), demonstrated significantly lower rates of CMV disease compared to prophylaxis. Other outcomes, including graft related outcome and mortality, were without significant difference [3].

Previous non-randomized studies have supported our findings, suggesting that prophylaxis is beneficial during its course, while merely delaying CMV disease to the post-prophylaxis period [20–24]. Singh et al. suggested that while using preemptive approach, low-level viremia induces an immune response, including specific cytotoxic T cells, later protecting against CMV disease. Such immune response is not induced while on prophylaxis, thus explaining the lower late-onset CMV disease in the preemptive arm [18].

Our finding of higher rates of acute kidney injury with prophylaxis was not demonstrated previously. Owers et al. reported higher leukopenia rates using prophylaxis [2].

A more recent meta-analysis included only kidney transplant recipients, and allowed prophylaxis with val/acyclovir. In this meta-analysis, similar findings of lower on-prophylaxis with higher late CMV infection rates were demonstrated, and leukopenia was significantly more common with prophylaxis. In addition, this meta-analysis evaluated cost to be similar between prophylaxis and preemptive approach [25].

Recent American society guidelines for the prevention of CMV disease suggest either preemptive approach or prophylaxis for most patients, considering appropriate resources for preemptive approach are available. For some D+R−patients, prophylaxis is recommended preferably. The guidelines also address the risk for post-prophylaxis CMV disease, suggesting several strategies in the 3–6 months after prophylaxis discontinuation. Close clinical monitoring of patients for CMV disease symptoms is suggested; virological monitoring could also be considered, though the optimal frequency and duration are unknown; and prolongation of prophylaxis could also be an option, though in the expense of adverse effect, as well as risk for later post-prophylaxis disease. Algorithms for prevention of CMV using immune monitoring, utilizing tests for cellular immunity to individualize duration of prophylaxis, are not yet fully defined in practice [8].

Our meta-analysis is limited by the low numbers of included trials, mainly the small number of trials conducted in recent years, complicated by the various types of organs transplanted (lung, liver, kidney). Accordingly, we could not address prophylaxis with drugs other than ganciclovir/valganciclovir. Neutropenia is associated with the use of val/ganciclovir as the prophylaxis agent, while novel antiviral medications such as letermovir are less myelosuppressive [26]. Another limitation is that only one trial reported lung transplant recipients [19] (Palmer), who are at highest risk for developing severe CMV disease.

In summary, using CMV prophylaxis after solid organ transplant resulted in lower rates of CMV infection and higher acute kidney injury rates with no difference in CMV disease at 12 months post-transplantation. Late-onset CMV disease rates were lower with preemptive therapy. No significant difference in overall mortality or graft related outcomes were documented. CMV preemptive approach is reasonable for CMV prevention in SOT recipients, if logistically and economically feasible. Optimal viral load cutoff values for therapy and optimal frequency of monitoring should be further explored. Strategies for combining the two approaches, as well as immune monitoring, should be investigated.

## Supplementary Information

Below is the link to the electronic supplementary material.

Supplementary file1 (DOCX 13 KB)

Supplementary file2 Mortality (TIFF 141 KB)

Supplementary file3 CMV syndrome (TIFF 139 KB)

Supplementary file4 Graft rejection (TIFF 141 KB)

Supplementary file5 Graft loss (TIFF 117 KB)

## Data Availability

No datasets were generated or analysed during the current study.
