# Supplementary material for: Prophylactic vs preemptive strategy for the prevention of CMV disease in solid organ transplant recipients: systematic review and meta-analysis of randomized controlled trials
Source: Infection. 2024 Nov 22;53(3):1091–9. doi: 10.1007/s15010-024-02441-4 (PMC12137393; doi:10.1007/s15010-024-02441-4)
Supplement: Supplementary file 1 — Supplementary file1 (DOCX 13 KB) [file 15010_2024_2441_MOESM1_ESM.docx]

Supplemental appendix

Search Term by database

PubMed:

("CMV"[Title/Abstract] OR "cytomegalovirus"[Title/Abstract]) AND (Organ Transplant* OR Heart Transplant* OR Kidney Transplant* OR Renal Transplant* OR Liver Transplant* OR Lung Transplant* OR Pancreas Transplant*) AND ((randomized controlled trial[pt] OR controlled clinical trial[pt] OR randomized[tiab] OR placebo[tiab] OR clinical trials as topic[mesh:noexp] OR randomly[tiab] OR trial[ti] NOT (animals[mh] NOT humans [mh]) OR RCT[Title/Abstract] OR randomised controlled trial[Title/Abstract] OR randomized controlled trial[Title/Abstract]))

Cochrane and Web of Science:

(Organ transplat* OR Heart Transplant* OR Kidney Transplant* OR Renal Transplant* OR Liver Transplant* OR Lung Transplant* OR Pancreas Transplant*) AND (CMV or cytomegalovirus) AND (Randomized controlled trials OR Randomised controlled trials)
