# Supplementary figures and images for: Prophylactic vs preemptive strategy for the prevention of CMV disease in solid organ transplant recipients: systematic review and meta-analysis of randomized controlled trials
Source: Infection. 2024 Nov 22;53(3):1091–9. doi: 10.1007/s15010-024-02441-4 (PMC12137393; doi:10.1007/s15010-024-02441-4)

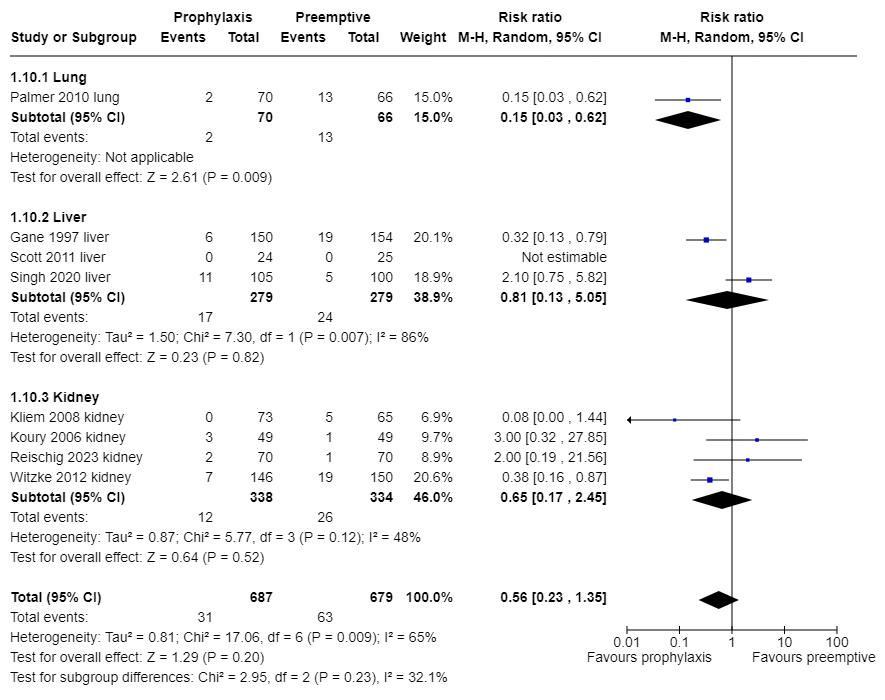

Supplement: Supplementary file 2 — Supplementary file2 Mortality (TIFF 141 KB) [file 15010_2024_2441_MOESM2_ESM.tiff]

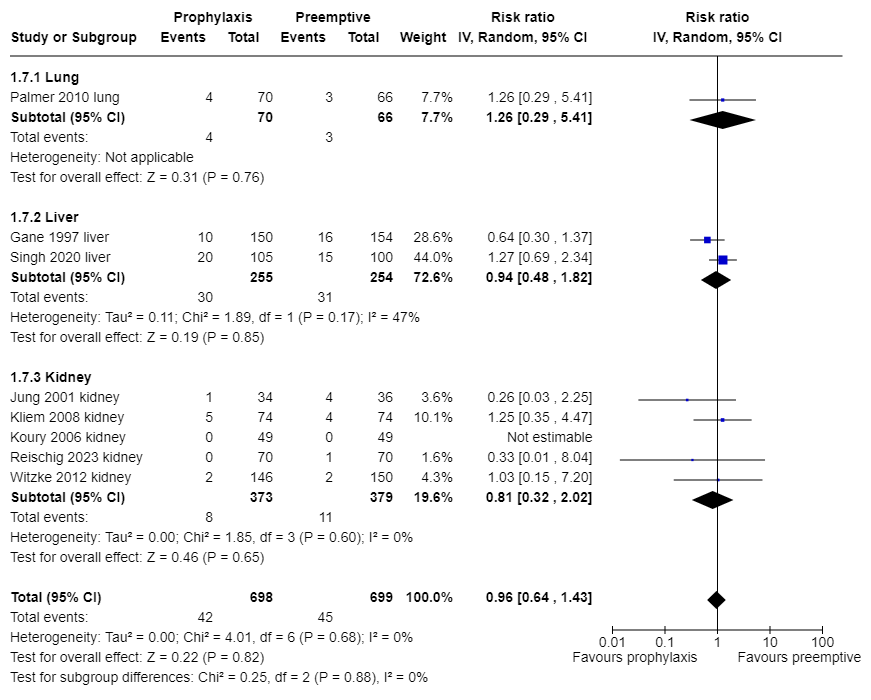

Supplement: Supplementary file 3 — Supplementary file3 CMV syndrome (TIFF 139 KB) [file 15010_2024_2441_MOESM3_ESM.tiff]

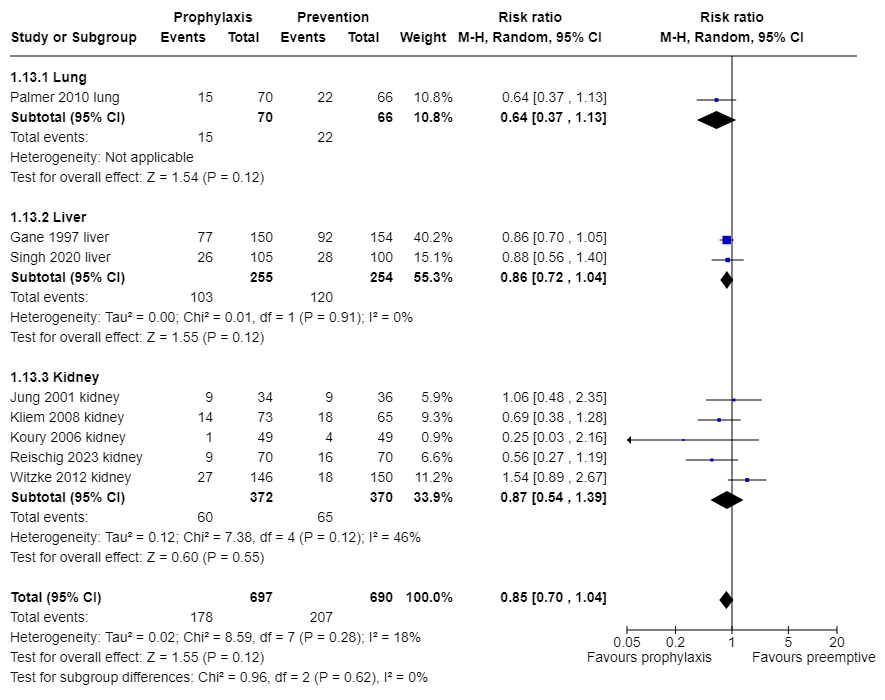

Supplement: Supplementary file 4 — Supplementary file4 Graft rejection (TIFF 141 KB) [file 15010_2024_2441_MOESM4_ESM.tiff]

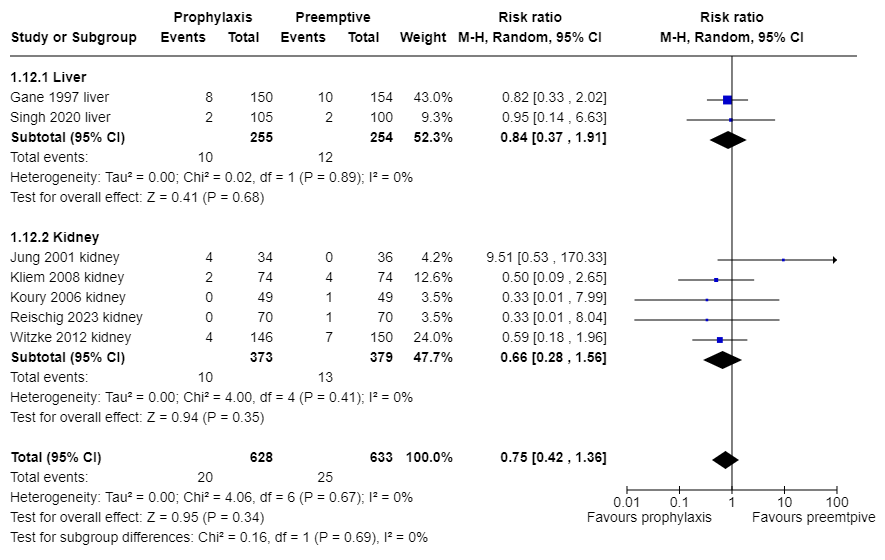

Supplement: Supplementary file 5 — Supplementary file5 Graft loss (TIFF 117 KB) [file 15010_2024_2441_MOESM5_ESM.tiff]
